# Clinical and radiological outcome of minimally invasive posterior lumbar interbody fusion in primary versus revision surgery

**DOI:** 10.1186/s13018-015-0337-y

**Published:** 2016-01-04

**Authors:** B. Hentenaar, A. B. Spoor, J. de Waal Malefijt, C. H. Diekerhof, B. L. den Oudsten

**Affiliations:** Department of Orthopedics, Clinical Orthopedic Research Center (CORC-mN), Diakonessenhuis Utrecht/Zeist, Bosboomstraat 1, 3582 KE Utrecht, The Netherlands; Department of Orthopedic Surgery, St Elisabeth Hospital, Hilvarenbeekse weg 60, 5000, LC Tilburg, The Netherlands; Department of Medical and Clinical Psychology, St Elisabeth Hospital, Hilvarenbeekse weg 60, 5000, LC Tilburg, The Netherlands

## Abstract

**Purpose:**

The aim of this study is to compare the clinical and radiological outcome of minimally invasive posterior lumbar interbody fusion (MI-PLIF) in revision and primary cases.

**Methods:**

In a retrospective study, we compared the clinical and radiological results of MI-PLIF for *lytic spondylolisthesis* (*n* = 28) and *recurrent radiculopathy after herniated disc surgery* (*n* = 28). Clinical outcome was assessed using the visual analogue score (VAS) and Oswestry Disability Index (ODI). Quality of life was assessed with the Euroqol-5d (EQ5D), the EQ5D VAS and the WHOQOL-BREF.

**Results:**

The follow-up was 5.1 (SD 2.3) years. The decrease in VAS scores was significant and comparable in both groups. We found significantly better ODI and quality of life scores for the patients with lytic spondylolisthesis. The radiological outcome showed only one non-union, and subsidence occurred in both groups at an equal amount.

**Conclusion:**

The MI-PLIF technique is a safe procedure with only few complications and a high fusion rate. It was successful in both groups, but the quality of life and ODI are better in primary cases.

## Introduction

The use of minimally invasive surgery (MIS) techniques for lumbar interbody fusion has become very popular in the last decade. Percutaneous lumbar pedicle screw insertion was first reported by Magerl in 1982 in combination with external fixation of the spine [1]. In 1995, Matthew and Long used percutaneous pedicle screws in combination with plates as an internal fixation system in the subcutaneous tissue in order to reduce the risk of infection, but the relatively superficial implants made this technique poorly tolerated [2]. Foley et al. subsequently revised the instrumentation in order to allow subfascial placement of the rods. Advances in the design of percutaneous pedicle screws, combined with the tubular retractor system, led to the development of minimally invasive posterior lumbar interbody fusion (MI-PLIF). In 2001 and 2002, Foley et al. were the first to describe their results about MI-PLIF in 12 patients, who were followed for an average of 13.8 months. Six patients were rated with excellent results, five patients with good results and one with a poor result [3, 4].

Since 2002, several clinical series have been published using different percutaneous techniques for lumbar interbody fusion. They report good short-term results with minimization of blood loss and soft tissue trauma, leading to shorter hospitalization and faster recovery compared to the traditional open procedures [5–7]. In our hospital, we use the MI-PLIF technique mostly for patients with lytic spondylolisthesis and patients with recurrent leg pain after previous herniated disc operations, often referred to as failed back surgery syndrome (FBSS). FBSS is an umbrella concept and has no agreed-on definition. We prefer to use the term *recurrent radiculopathy after herniated disc surgery*.

Lumbar fusion in patients with lytic spondylolisthesis is successful and shows good to excellent clinical outcome in generally above 80 % of the cases using different methods [7–11]. The results of lumbar fusion in patients with FBSS are less predictable. This is a heterogeneous group with often multiple previous operations and a long history of pain. Factors that affect surgical outcome are numerous, ranging from patient psychosocial factors to the technical aspects of a revision procedure [12, 13].

We assume that patients with a lytic spondylolisthesis have a better clinical outcome and quality of life than patients with recurrent radiculopathy. To our knowledge, there is no study comparing the outcome of lumbar fusion surgery in these two patient groups.

## Patients and methods

### Study design

In our hospital, we performed MI-PLIF (CD Horizon Sextant, Medtronic Sofamor Danek, Memphis, TN, USA) in 269 adult patients (age >18 years) between April 2002 and December 2009. From these 269 patients, a one-level interbody fusion with decompression was performed in 60 patients for grade I or II spondylolytic spondylolisthesis (Meyerding classification) in L4 or L5. Forty out of these 60 cases did not have any previous spinal operations and did not suffer from other spinal pathologies. In the group of 269 patients, 163 patients underwent a MI-PLIF at one or two levels because of recurrent radiculopathy after previous herniated disc surgery. Forty-six patients were operated on because of other diagnosis like fractures, metastases, multilevel pathology without prior surgery or a prior lumbar spondylodesis.

**Fig. 1:**
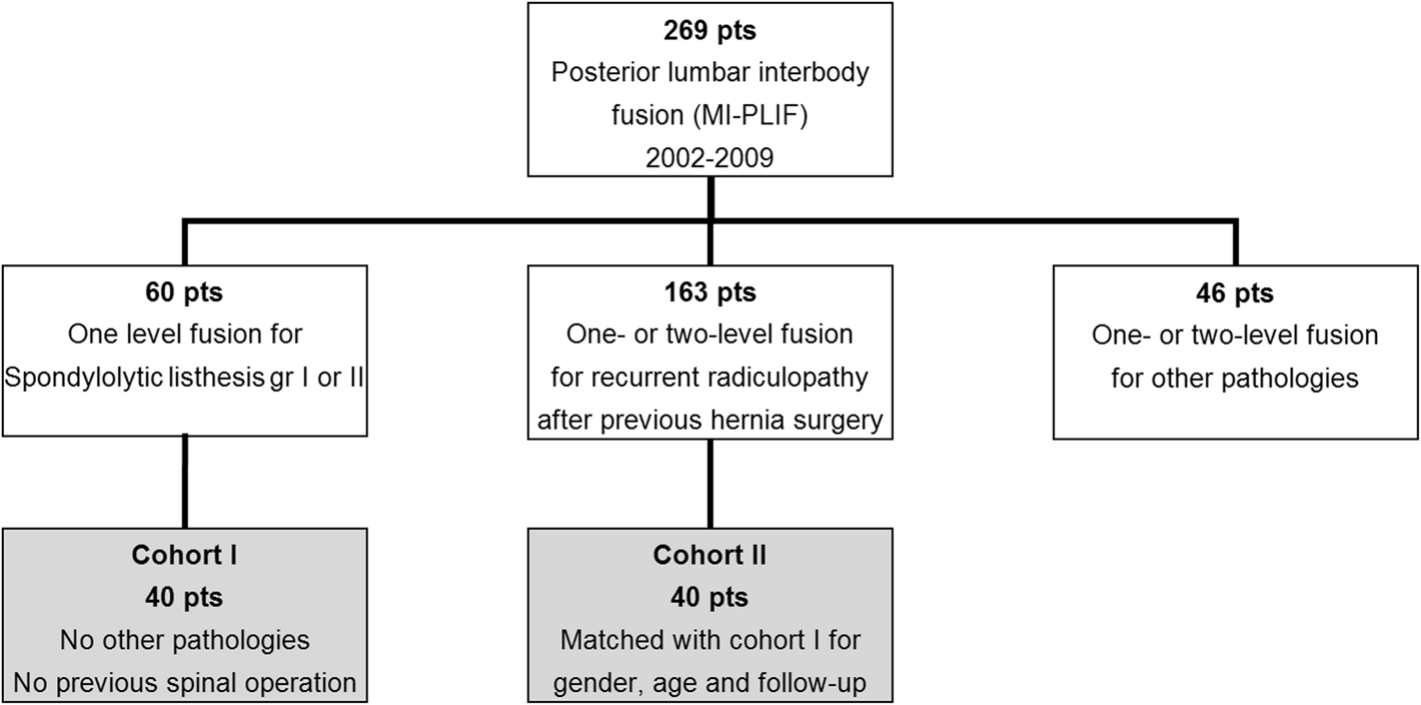
Patient selection

The first cohort of 40 patients with lytic spondylolisthesis was matched with 40 patients from the group with recurrent radiculopathy after hernia surgery. All patients were matched with a patient of the same gender who underwent surgery within a maximum time frame of 6 months before or after the patient in cohort I. In this time window, the person with the best age resemblance was selected. Then we had two matched cohorts (I and II) of 40 patients (Fig. 1). We invited all patients by means of a letter and an additional phone call to participate in our study. Patients were asked to fill in the questionnaires for the WHOQOL-BREF, Euroqol-5d (EQ5D), Oswestry Disability Index (ODI) and visual analogue score (VAS) for back and leg pain. A preoperative VAS score was present in most patients, and demographic data were collected from the medical records (age, gender, length, weight, ASA classification and smoking habits).

Ethical approval for our study was given by the METC Brabant (email: info@metcbrabant.nl) with *METC number: 1202* and *NL number 38113.008.11*.

### Operative technique

All patients were operated by two orthopaedic surgeons (CHD and JWM), and neural decompression was performed together with one of the neurosurgeons in our hospital. Fluoroscopy was used to check the appropriate level. A 3- to 5-cm midline skin incision was made. The fascia was incised and the paravertebral muscles dissected from the spine. Neural decompression was performed by bilateral laminectomy, a complete annulotomy and discectomy. Two cages were placed for every fusion level (Capstone interbody device, Medtronic Sofamor Danek, Minneapolis). A local bone obtained during decompression was morselized and impacted in the cages. The right position of the cages was confirmed under fluoroscopy. Pedicle screws were placed with the use of computer navigation (StealthStation fluoroscopic navigation system, Medtronic). In case of a two-level fusion, four pedicle screws were used, two in the most proximal and two in the most distal pedicles.

### Radiographic assessment

**Fig. 2:**
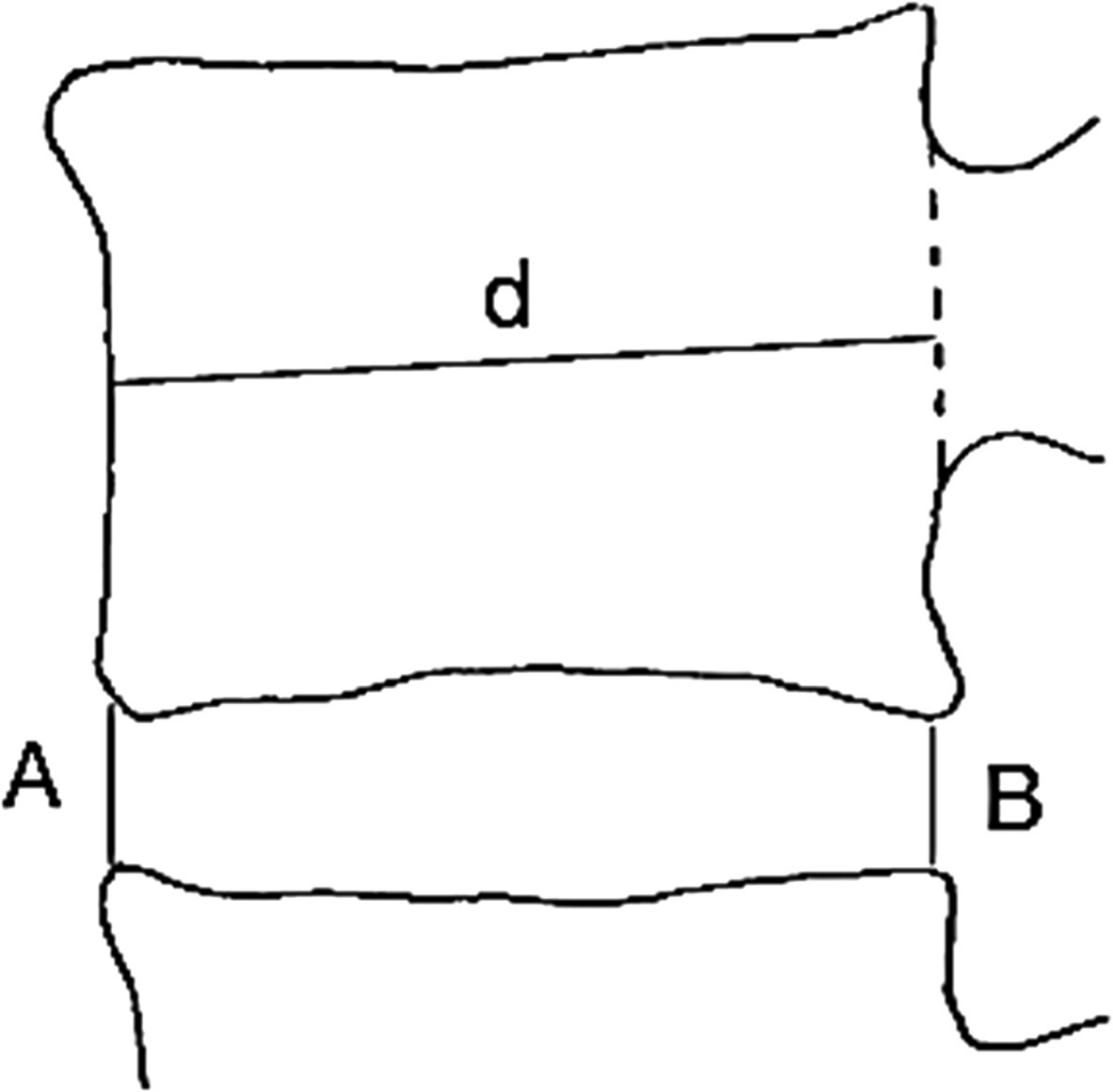
Disc height index according to Dabbs’ method. The average intervertebral disc height (*A+B/2*) was normalized with the anteroposterior diameter (*d*) of the upper vertebral body to correct for the magnification differences of the radiographs

In all patients, a lateral upright lumbosacral radiograph was made in our radiology department. In order to calculate the amount of subsidence, the intervertebral disc height index (DHI) was measured preoperatively, postoperative and at last follow-up in all patients according to the Dabbs’ method [14, 15]. The intervertebral disc height is expressed as an average of the sum of the measurements at the anterior and posterior regions of the disc (A+B/2). The disc space height is normalized with the anteroposterior diameter (*d*) of the upper vertebral body to correct for the magnification differences of the radiographs (Fig. 2). The average DHI per fused lumbar level was compared in the two cohorts. The subsidence was calculated as the difference in DHI between the postoperative and last follow-up X-ray. In addition, we searched for a correlation between the clinical outcome and the amount of subsidence. Further, we looked for signs of non-union like radiolucent lines around the implants or collapse. All radiographs were assessed and measured by the same observer (BH).

### Statistical analysis

Because of a non-parametric distribution of the cases in this study, we used the Mann-Whitney test for most of the variables (ODI, VAS, WHOQOL-BREF, weight, age and follow-up time) to compare the scores in both groups. For the EQ5D, ASA classification and smoking habits, we applied a chi square test. We used a repeated measures ANOVA for comparing the differences in VAS scores pre- and postoperatively. For comparison of the difference in DHI preoperatively, postoperatively and at last follow-up, we used a repeated measures ANOVA as well. For comparison of the difference in subsidence between both cohorts, we used a Mann-Whitney test. Statistical significance was set at a *P* value <0.05. All demographic and statistical analyses were performed using SPSS software (SPSS statistics 19, SPSS, Chicago, Ill, USA).

## Results

Sixty-four out of 80 patients were willing to participate in our study (33 cases in cohort I and 31 cases in cohort II), 4 patients could not be traced, 2 lived abroad, 1 demented and 9 did not want to participate because of various reasons (not spine-related immobility, too little time and simply no interest). All patients were seen in the outpatient clinic by the same observer (BH) in June 2012. Of these 64 patients, there were 28 matched pairs in both cohorts. Demographic data collected from both cohorts are listed in Table 1. None of the selected patients suffered from other causes of leg or back pain (like osteoarthritis of the knee/hip or peripheral arterial disease). There were no significant differences in demographic data between both groups, although the average weight, ASA classification and number of smoking persons were higher in cohort II. Moreover, the patients in cohort II suffered longer from back and/or leg pain prior to the lumbar fusion than the patients in cohort I, respectively, 8.79 (2 to 25) versus 7.64 (1 to 30) years. The complications in both cohorts are in Table 2. In both groups, one patient needed a reoperation because of malposition of one of the pedicle screws causing radicular pain immediately after the operation. In nine patients, the implants were removed because of persistent back pain. This was done at least 2 years after the operation, and in one of these patients (cohort II), there was a non-union of one of the two fused levels.

**Table 1:** Demographic data in cohorts I and II

	Cohort I	Cohort II
Age (years)	50.5 (33–76)	48.8 (37–76)
Gender (F:M)	1:1.9	1:1.9
Length (cm)	173 (152–190)	175 (156–195)
Weight (kg)	82.2 (57–120)	84.1 (57–128)
ASA	1.46 (1–2)	1.68 (1–3)
Follow-up (years)	5.1 (2–10)	5.1 (2–10)
Preoperative pain (years)	7.64 (1–30)	8.79 (2–25)
Smoking (%)	37	50
Previous operations of the lumbar spine	2.0 (1–15)	0.04 (0–1)

**Table 2:** ODI, EQ5D and WHOQOL-BREF (with four domains) in cohorts I and II

	Cohort I	Cohort II
ODI (%)	14.40 (0–70)	30.40 (0–70)
EQ5D	6.18 (0–9)	7.29 (0–11)
EQ5D VAS	77.90 (50–100)	62.07 (5–100)
WHOQOL-BREF	8.07 (4–10)	6.64 (2–10)
Physical health	15.46 (4–20)	13.00 (6–20)
Psychological health	16.07 (9–20)	15.57 (11–20)
Social relationships	16.61 (7–20)	15.67 (8–20)
Environment	17.29 (12–20)	15.00 (11–20)

The patients in cohort II had significant lower scores on most of the quality of life questionnaires. For the EQ5D, cohort I scored 6.18 (0–9) and cohort II scored 7.29 (0–11) points (*P* = 0.04). On the EQ5D VAS (Fig. 4), ranging from 0 to 100 with 100 indicating the best health a patient can imagine, patients in cohort I gave themselves 77.90 (50–100) points and in cohort II only 62.07 (5–100) points; this was also a significant difference (*P* = 0.02). For the WHOQOL-BREF, patients in cohort I scored significantly better for the overall quality of life (*P* = 0.002) and for the domains physical health (*P* = 0.008) and environment (*P* = 0.002). The domains social relationships and psychological health did not show a significant difference (*P* > 0.05). The results of the health scores are in Table 2.

**Fig. 3:**
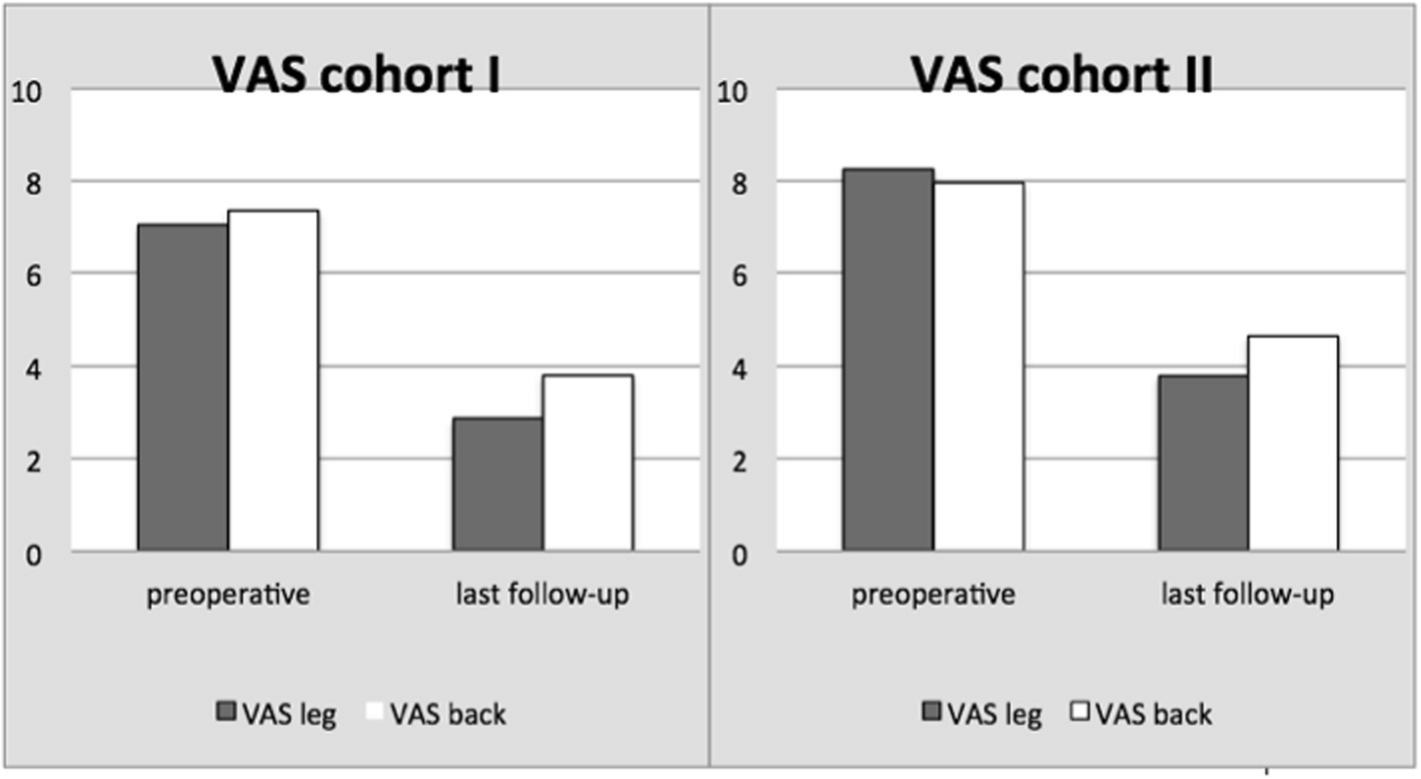
VAS pain scores for leg and back in cohorts I and II

**Fig. 4:**
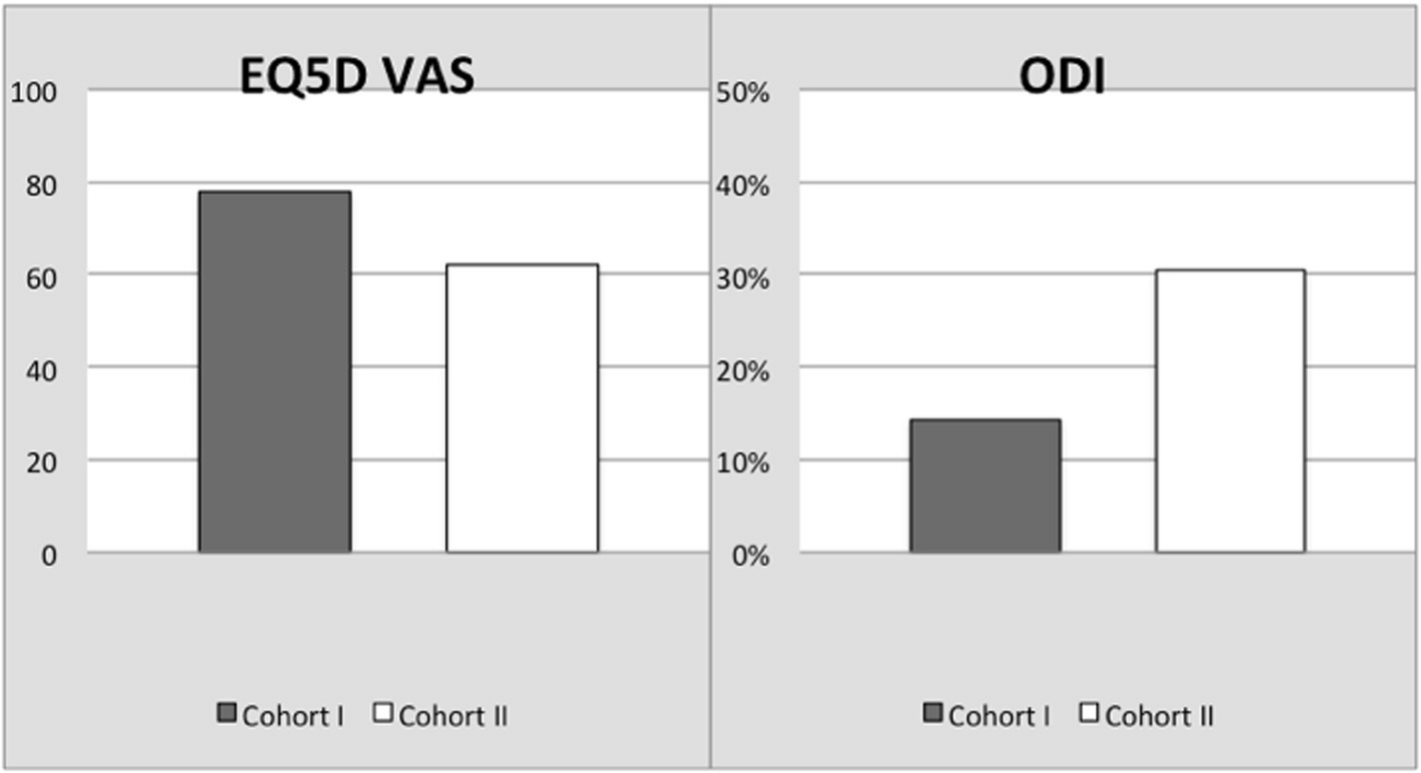
EQ5D VAS and Oswestry Disability Index in cohorts I and II

The pain and disability scores are shown in Figs. 3 and 4. The ODI showed a significant difference (*P* = 0.003) and was better in cohort I with 14.40 % (minimal disability) than in cohort II with 30.40 % (moderate disability); see Table 2. The decrease in VAS score for leg and back pain was significant in both groups (*P* < 0.001) comparing the VAS preoperatively with the VAS at last follow-up. The patients in cohort I had a lower VAS score for both leg and back pain than the patients in cohort II prior to the operation as well as at the last follow-up (Fig. 2). Nevertheless, this difference between the cohorts was not significant (*P* > 0.05). We also looked for a difference in the amount of change between VAS scores pre- and postoperatively. Both cohorts had the biggest decrease in VAS score for the leg pain; this was 4.18 (0–10) points in cohort I and 4.32 (0–10) points in cohort II. The back pain decreased 3.57 (0–9) in cohort I and 3.32 (0–9) in cohort II. These differences were not significant (*P* > 0.05).

**Fig. 5:**
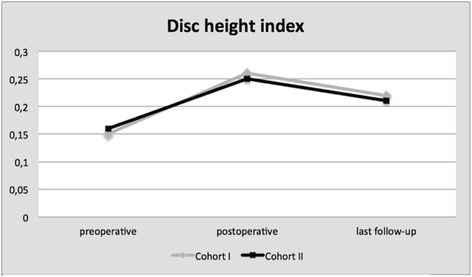
Disc height index per fused lumbar level in cohorts I and II

The DHI was calculated for every operated lumbar level separately preoperatively, postoperatively and at last follow-up. The values in both cohorts are more or less equal (Fig. 5). As expected, we found a significant (*P* < 0.001) increase in the DHI directly postoperatively in comparison to the preoperative situation. For cohort I, we found a subsidence of 0.04 (0–0.11) and for cohort II of 0.05 (0–0.18). Despite this amount of subsidence, there was still a significant gain in disc height at the last follow-up compared to the preoperative situation (*P* = 0.02). The difference in subsidence between cohorts I and II was not significant (*P* > 0.05). We could not find any correlation between cases with more subsidence and cases with the worse clinical outcome.

In none of the cases, signs of loosening like collapse of the pedicle screws, bars or cages were found. One patient in cohort II showed a non-union on the X-ray at the last follow-up (Table 3). In all other cases, the X-rays showed bony fusion of the operated levels.

**Table 3:** Complications in cohorts I and II

	Cohort I	Cohort II
Infection	1	0
Reoperation	1	1
Non-union	0	1
Implant removal	3	6
Other	1	2

## Discussion

*Lytic spondylolysis* and *recurrent radiculopathy after herniated disc surgery* are both common indications for lumbar fusion. Whereas the first indication is known for the most predictable and good results with different surgical techniques [7–9, 11, 16, 17], the latter has a less predictable outcome with sometimes disappointing results [12, 13]. The advantages of the patients in our first cohort are obvious: a clear indication, shorter history of complaints and a primary operation in untouched tissue with clear landmarks. The second cohort is much more challenging with a difficult indication, multiple previous operations, chronic pain and altered anatomy. Further, psychosocial factors may strongly influence the outcome in the second cohort [12]. The best technique to achieve lumbar interbody fusion in both pathologies is unknown.

MIS has become very popular in the last decade. It minimizes soft tissue injury and has less blood loss, faster recovery and shorter hospital stay [11, 18–20]. The drawbacks to MIS are the longer learning curve, higher dose of radiation, longer surgery time and increased risk of technical failure [5–7, 19]. Results are comparable with the open counterparts concerning the decompression and consolidation, although there are only few trials comparing both techniques [5–7]. Little has been published about the use of MIS techniques for revision operations of the lumbar spine. Selznick et al. compared 17 revision cases to 26 primary lumbar interbody fusions with MIS (TLIF and PLIF) and found no difference in blood loss or neurologic comorbidity. However, they did find a higher rate of incidental durotomy in the revision group. They conclude MIS to be technically feasible for revision operations, but it demands significant experience [20]. We did not look for the number of dural tears in this study, but overall, the complication rate in both cohorts was similar (Table 3). The data on MIS for the treatment of lytic spondylolysis are accumulating [7, 9, 11, 19]. Park et al. published a series of 40 patients with a mean follow-up of 35 months who underwent MI-TLIF for spondylolisthesis in combination with decompression and reduction of the anteroposition by a reduction screw extender. They conclude MIS for spondylolisthesis to be an effective option, yielding clinical outcomes that are comparable to open surgical procedures [11]. Wang et al. reported a prospective randomized trial of MI-TLIF versus an open TLIF in 85 cases for spondylolisthesis. They found a significant improvement for VAS and ODI in both groups but no significant difference between both techniques. No difference was found in the number of complications [7]. Laminar decompression for spondylolisthesis in case of nerve root pain seems to be beneficial, but evidence is lacking [8, 16]. Benefits of reduction of the listhesis also have not been adequately studied. From the few studies that report the preoperative and postoperative degree of the listhesis, it appears that low-grade (<50 %) listhesis can be reduced to some extent, but complete reduction is rarely achieved or maintained [8, 11, 17].

This study shows the results of 56 patients undergoing a MI-PLIF for *lytic spondylolisthesis* or *recurrent radiculopathy after herniated disc surgery*. Both groups showed significant improvement in VAS and ODI scores. This improvement is comparable with other studies on patients undergoing lumbar fusion surgery for lytic spondylolisthesis [7, 11, 19] as well as for recurrent radiculopathy after herniated disc surgery [20–22]. With a minimum follow-up of 30 months, this symptomatic improvement appears durable. Against our expectations, we did not find a significant difference for the VAS scores between the cohorts. The ODI was significantly better in cohort I, supporting our assumption. Most studies describe better postoperative VAS scores for leg pain than for back pain [11, 21, 22] as is similar in our patients (Fig. 4).

The perceived physical and mental health was much worse for patients in cohort II. They had significant lower scores for the EQ5D, EQ5D VAS and two out of four domains of the WHOQOL-BREF (Table 2). The objective physical health as measured with the ASA classification was also lower in cohort I than in cohort II, although this was not a significant difference (Table 1). The relation between the perceived clinical outcome and a possible underlying anxiety disorder or depression is known [12, 21]. Although the patients in cohort II were significantly more negative about their physical health and quality of life, they showed a significant improvement in VAS and ODI. This confirms our experience that you can have satisfactory results with MIS in lumbar revision surgery with careful patient selection and good preoperative counselling on patient expectations and surgical success, as stated before in other publications [12, 13, 21].

In the studies on lumbar interbody fusion, many kinds of cages are used. Differences in migration, subsidence, lordosis and fusion rate have not been well studied. Many researchers have noted a gradual decrease of disc height due to cage subsidence and mention a possible adverse effect on the fusion rate [17, 22–24]. The decrease in disc space height does not seem to correlate with the clinical outcome [17, 22, 24]. Our patients showed a significant subsidence in both groups but only one pseudoartrosis after 5.1 (SD 2.3) years. We found no correlation between the degree of subsidence and the clinical outcome or radiographic fusion rate. We believe subsidence is the incorporation process of the cage to both endplates during the first months after surgery. It does not seem to affect fusion rate or clinical outcome.

This study contributes to the little literature about the use of MIS in revision cases or FBSS. There are no studies comparing the outcome of MIS in the context of primary versus revision surgery as we did with our two cohorts. Further, our follow-up represents the longest to date for studies describing the MI-PLIF technique. This study has also several limitations. First, the study is conducted as a small retrospective series with limited follow-up moments. Secondly, we did not measure the pre- and postoperative lumbar lordosis and pelvic parameters. The increase of lumbar lordosis and decrease of pelvic tilt may play an important role in better surgical outcome. A recent study showed that PLIF could restore the sagittal balance better than posterolateral fusion [25]. However, it is important to mention that the literature on lumbar fusion is primarily retrospective and heterogeneous with respect to indications, techniques and outcome measures. This makes it difficult to compare studies and draw conclusions.

## Conclusion

The MI-PLIF technique is a safe procedure with only few complications and a high fusion rate. It is successful for primary cases (*lytic spondylolisthesis)* as well as for revision cases (*recurrent radiculopathy after herniated disc surgery)* with a significant reduction in pain and disability. We hypothesized that we would not find a difference in the clinical and radiological outcome between both cohorts. For the radiological outcome, we did indeed find no difference in fusion rate or subsidence. For the clinical outcome, the primary cases scored significantly better in the ODI and quality of life scores, but not in the VAS scores.
